# *Musashi* expression in intestinal stem cells attenuates radiation-induced decline in intestinal permeability and survival in *Drosophila*

**DOI:** 10.1038/s41598-020-75867-z

**Published:** 2020-11-05

**Authors:** Amit Sharma, Kazutaka Akagi, Blaine Pattavina, Kenneth A. Wilson, Christopher Nelson, Mark Watson, Elie Maksoud, Ayano Harata, Mauricio Ortega, Rachel B. Brem, Pankaj Kapahi

**Affiliations:** 1 Buck Institute for Research on Aging, 8001 Redwood Boulevard, Novato, CA 94945 USA; 2 National Center for Geriatrics and Gerontology, 7-430 Morioka-cho, Obu, Aichi 474-8511 Japan; 3 SENS Research Foundation, 110 Pioneer Way, Suite J, Mountain View, CA 94041 USA

**Keywords:** Molecular biology, Physiology, Stem cells

## Abstract

Exposure to genotoxic stress by environmental agents or treatments, such as radiation therapy, can diminish healthspan and accelerate aging. We have developed a *Drosophila melanogaster* model to study the molecular effects of radiation-induced damage and repair. Utilizing a quantitative intestinal permeability assay, we performed an unbiased GWAS screen (using 156 strains from the *Drosophila* Genetic Reference Panel) to search for natural genetic variants that regulate radiation-induced gut permeability in adult *D. melanogaster*. From this screen, we identified an RNA binding protein, *Musashi (msi),* as one of the possible genes associated with changes in intestinal permeability upon radiation. The overexpression of *msi* promoted intestinal stem cell proliferation, which increased survival after irradiation and rescued radiation-induced intestinal permeability. In summary, we have established *D. melanogaster* as an expedient model system to study the effects of radiation-induced damage to the intestine in adults and have identified *msi* as a potential therapeutic target.

## Introduction

A typical mammalian cell encounters approximately 2 × 10^5^ DNA lesions per day^1^. External stressors, both chemical and radioactive, and internal factors such as oxidative stress, are the primary sources of DNA damage^2^. The inability to correct DNA damage results in the accumulation of harmful mutations, which contribute to cellular damage, cancer, and aging^3–8^. However, DNA damaging agents, such as radiation, are the only available treatments for certain pathologies. These therapies can lead to complications due to cellular and tissue damage caused by genotoxic stress. For example, genetic and epigenetic alterations in the tumor^9^, or tumor microenvironment, may render it resistant to radiation^10^. Additionally, bystander tissues are also damaged from radiotherapy^11,12^. Patients undergoing radiotherapy encounter both short-term side effects (nausea, vomiting, and damage to epithelial surfaces) and long-term side effects like enteritis^13^, radiation proctitis, heart disease^14^, and cognitive decline^15^.

Multiple organisms have developed several DNA error correction mechanisms, as the inability to correct DNA errors leads to permanent cellular damage^16–19^. Cells undergo one of the following fates: apoptosis, replicative arrest, such as senescence, or clearance by phagocytosis or autophagy^20^. These fates often involve the cell non-autonomous interactions, which cannot be recapitulated by in vitro models of genotoxic stress. Furthermore, they fail to represent the complexities of tissue microenvironments and the cell non-autonomous consequences of radiation damage. For instance, apoptotic or senescent cells may produce secreted factors that exacerbate damage to cells that did not receive the primary insult^21^.

As the gastrointestinal tract encompasses a large area in the body and has the highest turnover rate^22,23^, it is commonly a bystander tissue in radiotherapy accounting for significant side effects of radiation treatment^24,25^. The fly and human intestines share similar tissue, anatomy, and physiological function^26,27^; both fly and mammalian guts are composed of intestinal stem cells (ISCs), enterocytes (ECs), and enteroendocrine (EE) cells^28^. ISCs are involved in regenerative and tissue-repair processes^29,30^ in flies and mammals^31^. DNA damage to the ISCs leads to a reduced proliferative potential, which contributes to the pathogenesis of radiation enteritis in patients undergoing radiation therapy^32–35^. Previous studies have used the flies to study radiation damage, but these studies have been restricted to studying its impact during development^36,37^. Studies involving *D. melanogaster* have revealed conserved molecular pathways that maintain stem cell function, tissue repair, and homeostasis in the intestine^38–40^. Here, we have taken advantage of the flies’ genetic malleability, short lifespan, and complex tissue microenvironments to develop a whole-animal model to study therapeutic targets for radiation damage to the intestine.

## Results

### Ionizing radiation reduces survival and locomotion in *D. melanogaster*

**Figure 1:**
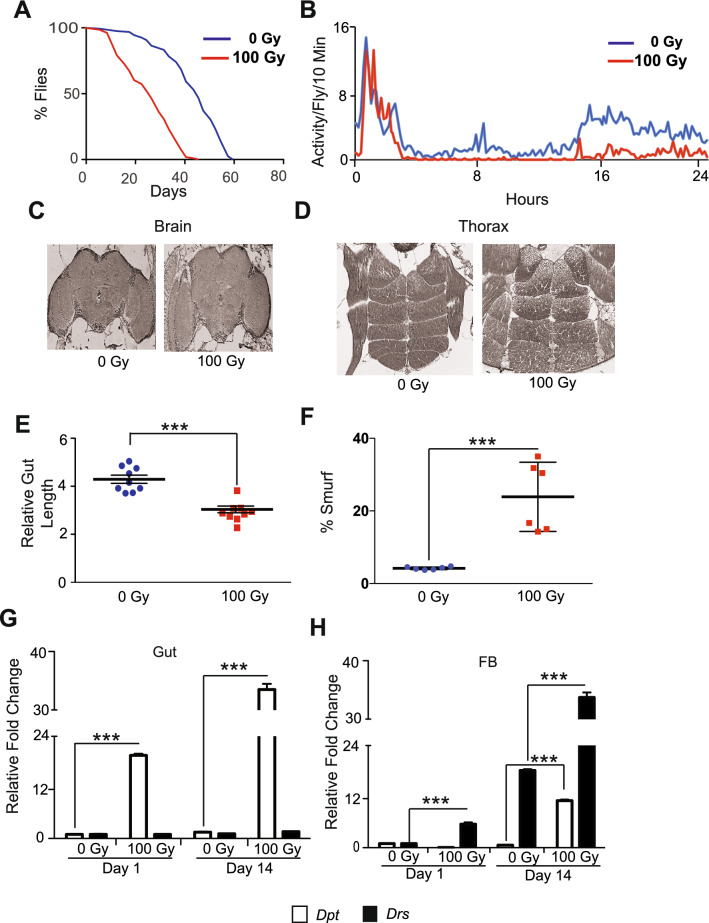
Radiation-induced damage in the gut results in a significant reduction in survival and spontaneous activity of *Drosophila*. (**A**) Kaplan Meier survival analysis upon irradiation of 5 day old flies. Non-irradiated control (0 Gy) and irradiated group (100 Gy), respectively. (**B**) The effect of radiation on spontaneous activity. The graph shows averaged activity per 10 min for control (0 Gy) and irradiated flies (100 Gy). The X-axis represents time (in hours) after the flies were moved to the activity monitors. The activity measurement was started at 4:00 p.m. (**C**) Representative H and E (Hematoxylin and eosin) staining of the paraffin-embedded brain and (**D**) thorax of *w*^*1118*^, 7 days after irradiation showing no structural abnormalities. (**E**) The graph represents intestine length measured using ImageJ. The relative length of the *w*^*1118*^ 5 days old adult female flies 14 days after irradiating with or without 100 Gy is plotted as arbitrary units. Each dot represents one sample. (***p < 0.001 by *t*-test). (**F**) The effect of radiation on gut permeability. Smurf assay to access gut permeability was performed in *w*^*1118*^ adult female flies 14 days after irradiating with or without 100 Gy. Results were plotted as mean percentage of ‘Smurf’ to non-smurf flies. Error bars indicate S.D. of 6 replicates. (***p < 0.001 by *t*-test). (**G**) Relative fold change in the expression of *Diptericin* (*Dpt*) and *Drosomycin* (*Drs*) in the gut and (**H**) fat body (FB). The results are represented as mean relative fold change in the gene expression and normalized to housekeeping gene *rp49* on days 1 and 14 after irradiation (100 Gy), demonstrating local and systemic inflammation. (***p < 0.001 by *t*-test).

Even though ionizing radiation (IR) is extensively studied in the context of mutagenesis experiments^41,42^ and embryonic development signals^43^ in *D. melanogaster,* not much is known regarding its effects in adult flies. We exposed 5-day old *w*^*1118*^ adult flies to different doses of IR. Interestingly, these flies were fairly resistant to lower doses of X-rays (from 1 to 10 Gy), likely because most tissues in the fly are post-mitotic^44^. However, when we exposed female *w*^*1118*^ flies to 100 Gy, it significantly reduced their mean lifespan, compared to un-irradiated controls (Fig. 1A and Supplemental Fig. 1A). We observed a similar reduction in lifespan in irradiated male flies, indicating that adult sensitivity to IR is sex independent (Supplementary Fig. 1B).

A frequent adverse effect of radiation exposure is fatigue^45–50^. Several groups have observed that irradiated mice have diminished spontaneous and voluntary activity^51,52^. We used the *Drosophila* Activity Monitor System to examine whether radiation also reduces flies' spontaneous physical activity^53^. Our results showed that irradiated flies display a reduction in spontaneous physical activity 14 days after IR exposure (Fig. 1B). To determine if this was due to damage to the brain and/or the muscles, we evaluated the morphological changes in the brain as well as flight and thoracic muscles in irradiated flies by hematoxylin and eosin (H & E) staining. We did not observe any overt structural damage to the brain (Fig. 1C). Also, we did not observe significant structural damage to the muscles in the thorax, day one (not shown), and seven after irradiation (Fig. 1D). These results indicate that neither muscle nor brain damage accounts for the reduction in survival and activity in irradiated flies.

### Ionizing radiation disrupts intestinal integrity and induces inflammation

Intestinal barrier disruption in flies is known to impact survival^54^. Thus, we examined whether the reduced survival of irradiated flies was due to damage to intestinal tissue. Our results showed that irradiated flies have significantly shorter intestines than non-irradiated controls 14 days after irradiation, which suggests that irradiation structurally damages the fly's intestine (Fig. 1E and Supplementary Fig. 1C). We hypothesized that this structural damage to the intestine influence barrier function. To test this, we measured the effect of irradiation on intestinal permeability by performing the previously described Smurf assay^55^, which involves feeding a blue dye. Our results in Fig. 1F demonstrate a significantly higher percentage of flies with permeable intestine (Smurf flies) upon irradiation when compared to un-irradiated controls 14 days after irradiation. We observed that this effect of ionizing radiation on intestinal permeability was responsive to increasing doses of radiation (Supplementary Fig. 1D). Furthermore, the cumulative effect of radiation on intestinal permeability when the dosage was staggered (4 doses of 25 Gy every other day) was similar in extent to the flies exposed to a single dose of 100 Gy, when intestinal permeability was measured by Smurf assay performed 14 days after irradiation (Supplementary Fig. 1E). The effect of exposure to ionizing radiation on intestinal permeability was sex independent, as Smurf assay revealed twofolds higher proportion of flies with permeable intestines in both males and females (Supplementary Fig. 1F). Finally, we also observed an increase in intestinal permeability in wild-type flies, *Canton-S*, due to damage caused by radiation (Supplementary Fig. 1G).

The disruption of gut barrier integrity after irradiation has been shown to result in increased local and systemic immune activation, indicated by the secretion of anti-microbial peptides (AMPs)^55^. To test this, we investigated the effect of radiation on the expression of the AMPs, *Diptericin (Dpt)* and *Drosomycin (Drs),* in dissected guts and fat bodies, which served as a proxy for systemic (fat bodies) and local (intestines) inflammation^56,57^. Quantitative realtime PCR (qRT-PCR) of RNA isolated from dissected intestinal tissue samples indicated a 20-fold increase in *Dpt* expression as early as 24 h after irradiation, which increased to 30-fold after 14 days of irradiation. This increase coincided with the intestinal permeability observed in our Smurf analysis (Fig. 1G), which also indicates elevated Immune Deficiency (IMD) signaling, a critical response to bacterial infection^58^ in the intestine of irradiated flies.

Fat body in *Drosophila* contributes to the humoral immune response. Hence its AMP production serves an indicator for systemic inflammation^59,60^. The qRT-PCR with the samples from the fat body revealed a fourfold increase in the expression of *Drs* after day 1, and a 35-fold increase 14 days after irradiation (Fig. 1H). This indicates a sustained increase in systemic inflammation from elevated Toll signaling^61^ in the fat body of irradiated flies. In the fat body we also saw an increase in the expression of *Dpt* by 14 days after irradiation. Together, these results demonstrate that irradiation induces a sustained local and systemic inflammatory response in the adult flies.

### Exposure to radiation caused DNA damage, cell death in enterocytes and inhibited ISC proliferation

**Figure 2:**
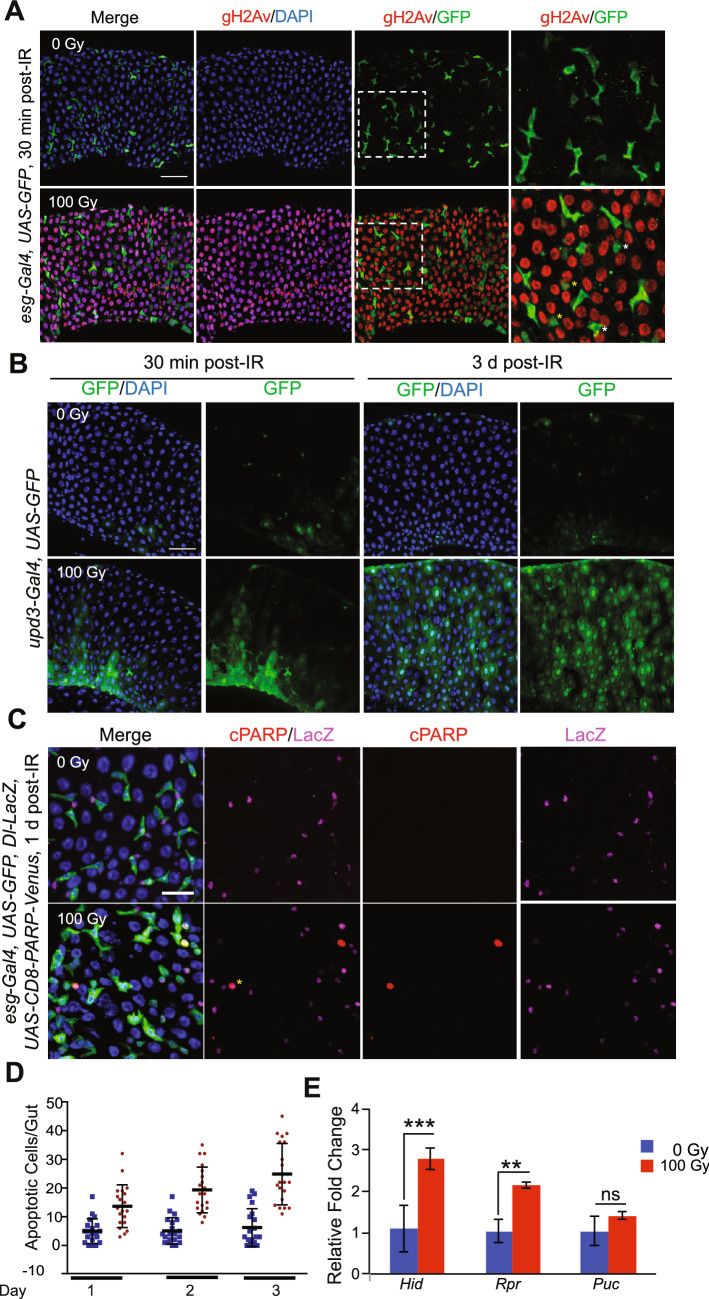
Exposure to radiation causes DNA damage and cell death in enterocytes and inhibition of ISC proliferation. (**A**) Midguts were stained with anti-γ-H2Av antibody and DAPI. Guts from *esg-Gal4, UAS-GFP* flies were dissected 30 min after irradiation with (bottom panels) or without (top panels) 100 Gy. Right panels are magnified images of the white square in the left side panels. Yellow asterisks indicate the both γ-H2Av and GFP positive cells. White asterisks indicate the γ-H2Av negative GFP positive cells. Scale bar indicates 40 µm. (**B**) Midguts were stained with anti-GFP antibody and DAPI. Guts from *upd3-Gal4, UAS-GFP* flies were dissected 30 min and 3 days after irradiation with (bottom panels) or without (top panels) 100 Gy. Scale bar indicates 40 µm. (**C**) Midguts were stained with anti-cPARP antibody, anti-LacZ antibody and DAPI. Guts from *esg-Gal4, UAS-GFP, Dl-LacZ, UAS-CD8-PARP-Venus* flies were dissected 1 day after irradiation with (bottom panels) or without (top panels) 100 Gy. Yellow asterisk indicates the both cPARP and LacZ positive cell. Scale bar indicates 20 µm. (**D**) Ethidium bromide-Acridine Orange staining was performed in guts of *w*^*1118*^ female flies after irradiation (100 Gy) at indicated time points. The results are plotted as the mean number of apoptotic cells per gut and presented and mean apoptotic cells and error bars indicate S.D. of 2 independent experiments with at least 10 guts each. (**E**) Relative fold change in the expression of *Hid, Rpr and Puc* in the dissected gut 24 h after irradiation. The error bars indicate S.D. of 4 replicates. (***p < 0.001, **p < 0.01, ^ns^p > 0.05 by *t*-test).

Exposure to ionizing radiation induces DNA double-strand breaks (DSB)^62,63^. One of the earliest events following DSBs is activation of kinases like ATM, ATR and DNA-PK, which phosphorylate the C-terminal tail of the histone 2A^64^. This DSB-induced phosphorylation of histone 2A (H2A) is conserved in *Drosophila*^65,66^. We tested the effect of ionizing radiation on histone γ-H2Av, the fly orthologue of H2AX, phosphorylation in flies' intestinal tissue by immunofluorescence staining. To visualize ISCs and its daughter cells, enteroblasts (EBs), we used *esg-Gal4* line to drive *UAS-GFP* transgene (referred to as *esg-GFP*)^28^. Following irradiation, cells in the intestine of the flies showed a substantial increase in γ-H2Xv foci compared to cells in the intestine of non-irradiated flies (Fig. 2A and Supplementary Fig. 2A). Although most of *esg-GFP* positive cells did not show γ-H2Xv foci, approximately 7% of these cells showed DNA damage (Fig. 2A and Supplementary Fig. 2A′). Environmental stress on gut enterocytes is known to activate reparative responses, often initiated by the IL-6-like cytokine, Upd3^67^. We tested if persistent DNA damage, caused by radiation, affects *Upd3* expression at 30 min and 3 days after irradiation. Our results indicated that the nuclear-localized *Upd3* expressing cells were significantly increased 3 days after irradiation, based on GFP reporter expression (Fig. 2B and Supplementary Fig. 2B).

Metazoan cells also undergo apoptosis following DNA DSB. Thus, we investigated the effect of radiation on apoptosis in the fly's intestine. We overexpressed *UAS-CD8-PARP-Venus*, a probe for the caspase activation^68,69^ in both ISCs and EBs using *esg-Gal4* with *Delta-LacZ* background, and apoptotic cells were detected by immunostaining with the anti-cleaved PARP antibody. We found that *Delta-LacZ* marked ISCs underwent apoptosis 1 day after irradiation (Fig. 2C and Supplementary Fig. 2C). These results suggested that increased gut permeability after irradiation is due to loss of regenerative capacity caused by the death of ISCs. SYTOX staining for apoptotic cells performed in these flies demonstrated *esg*-negative cells (EC, EE) are also becoming SYTOX positive after irradiation and undergoing apoptosis (Supplementary Fig. 2D and 2D′). We also performed Acridine Orange/Ethidium Bromide staining assay in dissected intestinal tissue, which showed a nearly two-fold increase in the number of apoptotic cells on days 1, 2 or 3 following irradiation^70^ (Fig. 2D). These results were also supported by increased expression of the pro-apoptotic genes, *hid* and *reaper*^71^ 24 h after irradiation as measured by qRT-PCR of RNA isolated from dissected intestine (Fig. 2E). Interestingly, we did not see an increase in the expression of *puckered*^72^, a marker of JNK induced apoptosis in the intestines of irradiated flies.

### Exposure to X-rays inhibited ISC proliferation and increased intestinal permeability

**Figure 3:**
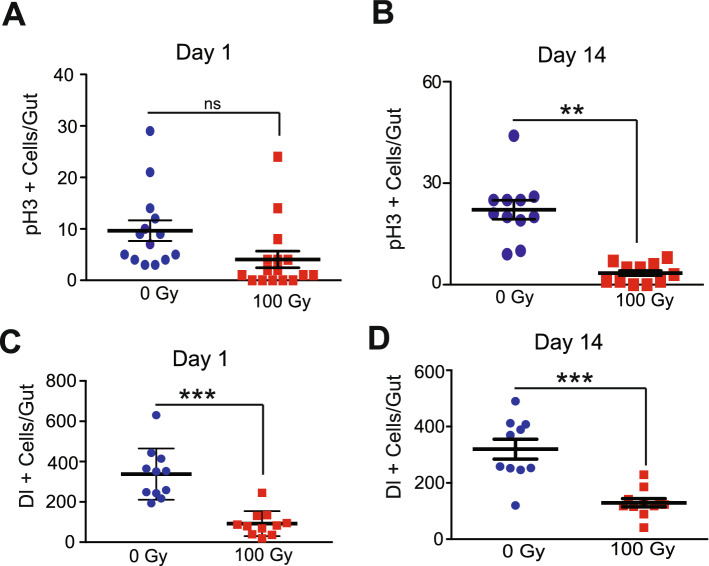
Radiation-induced damage inhibits the proliferation of intestinal stem cell. (**A**) ISC proliferation was measured by counting the numbers of pH3-positive cells detected in *w*^*1118*^ flies irradiated with 100 Gy day 1 and (**B**) day 14. The result is presented as mean ± SE of at least 10 guts per group. (**p < 0.01, ^ns^p > 0.05 by *t*-test). (**C**) The effect of irradiation on the ISC number was tested by counting the numbers of *Dl-LacZ* positive cells. ISC numbers were determined by immunostaining with anti-β-Gal antibody in dissected guts of *Dl-LacZ* flies on days 1 and (**D**) day 14 after irradiation. The result is presented as mean ± SE of at least 10 guts per group. (***p < 0.001 by *t*-test in each group). Error bars indicate SEM (***p < 0.001 by *t*-test).

Previous studies have shown that fly guts respond to damage from toxins like dextran sulfate sodium (DSS) or Bleomycin^73^, and stress from a bacterial infection^74^ by inducing the proliferation of ISCs which enhances intestinal repair by replacing damaged cells^75^. We investigated whether ISCs in irradiated flies could mediate tissue homeostasis by replacing apoptotic enterocytes by immunostaining guts with an anti-phospho-Histone H3 (anti-pH3) antibody that marks dividing cells. Immunofluorescence staining in dissected guts demonstrated that irradiation inhibited ISC proliferation as early as 1 day after irradiation (Fig. 3A). This inability of ISCs to repair damage was more clearly observed 14 days after irradiation (Fig. 3B). We also irradiated a fly strain harboring a *Delta-LacZ* enhancer trap that has been extensively used to identify ISCs^76,77^. We observed that exposure to radiation significantly reduced the number of *Delta* positive ISCs and ISC markers (1 and 14 days after irradiation) (Fig. 3C,D), consistent with our observation that *Delta* positive ISCs underwent apoptosis (Fig. 2C and Supplementary Fig. 2C).

### Fly Genome-Wide Association Study (GWAS) for radiation-induced intestinal permeability

Genetic variations influence sensitivity to genotoxic stress, the detrimental effects of radiation treatment, and the prognosis of radiation therapy^78–87^. We leveraged the *Drosophila* Genetic Reference Panel (DGRP) that contains flies with fully sequenced genetic variations, to conduct an unbiased GWAS screening of approximately 156 fly strains from the DGRP. Approximately 100 flies from each strain were irradiated with 100 Gy, and the percentage of Smurf flies was measured 14 days after irradiation. The genetic markers with > 25% minor allele frequency were used for screening^88^. The lines were split into two groups, one for each allele at a given genetic locus. Linear regression modeling was used to determine the difference between phenotypes associated with each allele. The FDR for each trait was calculated by permutation of the phenotypic data^89^.

**Figure 4:**
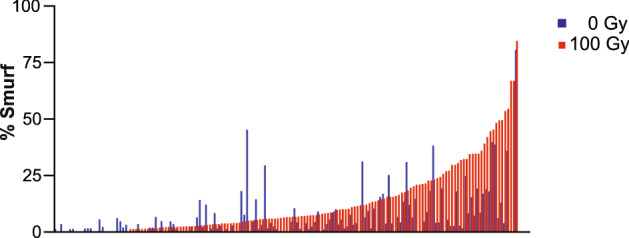
Phenotypic variation in gut permeability across 156 DGRP lines caused by radiation exposure*.* Lines are arranged in order of increasing phenotype of the irradiated lines, paired with their non-irradiated controls. Gut permeability was determined by Smurf assay and results were plotted as mean proportion of ‘Smurf’ to non-smurf flies in each group with at least 100 flies were tested per condition.

The DGRP lines varied in radiation-induced gut permeability, from a 14-fold increase in Smurf incidence to a 20-fold decrease in Smurf incidence (Fig. 4). Our GWAS analysis revealed several potential candidate genes (Table 1). However, we set a cutoff of false detection rate (FDR) of 27% or less to consider the genes for further validation^88^. We investigated the candidates listed in Table 1 for their ISC-specific influence on intestinal permeability after irradiation. To test this, we crossed fly lines expressing an RNAi against the candidate genes (like *msi*, *Ddr,* and *cka*) with lines expressing the drug (RU486)-inducible ISC-specific *5961-Gene Switch-Gal4* (*5961-GS*) driver and measured intestinal permeability 14 days after irradiation. We observed the most significant increase in gut permeability in *5961-GS* > *msi *^*RNAi*^(*musashi)* flies after irradiation (Supplementary Fig. 3).

**Table 1:** List of candidate genes identified by GWAS analysis of Smurf data collected from DGRP fly lines.

Cka marker	Gene	Human orthologue	Effect/location	Interaction P value	FDR (%)
2L_6284412_SNP	Ddr	DDR2	INTRON	0.00030849	0
2L_6283921_SNP	Ddr	DDR2	INTRON	0.00070189	8
3L_3480865_SNP	CG42324	TJAP1	INTRON	0.00109696	10
3R_21373234_SNP	msi	MSI1/2	INTRON	0.00140678	14
X_12489073_SNP	CG1824	ABCB8	NON _CODING	0.00206947	24
2L_8035397_SNP	Cka	STRN3	INTRON	0.00217694	25

The table is showing the location of the SNP associated with increased intestinal permeability, and location of the SNP. The candidates with False discovery rate ≤ 25% for analysis.

### *msi* regulated ISC function, intestinal permeability, and survival in response to radiation-induced damage

**Figure 5:**
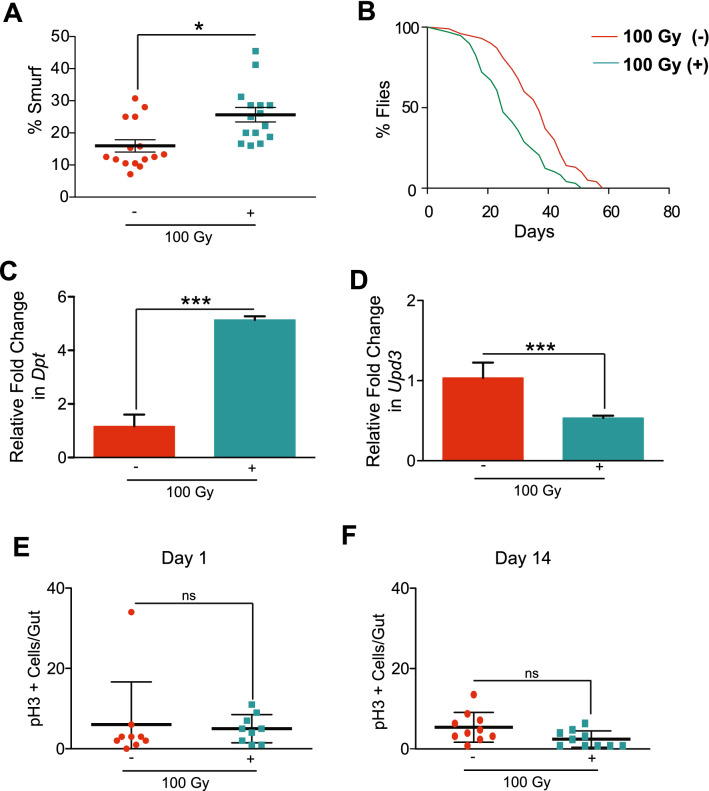
Reducing *msi* expression in ISCs increases gut permeability and reduces survival by inhibiting stem cell proliferation. (**A**) Smurf assay for assessing gut permeability was performed with *5961-GS* > *UAS-msi *^*RNAi*^ flies on day 14 after irradiation. Control 100 Gy (−) maintained without RU486, whereas *msi* was reduced in ISCs in 100 Gy (+). The error bars indicate S.D. of percent Smurf flies per vials. (*p < 0.05 by *t*-test). (**B**) Kaplan Meier survival analysis of *5961-GS* > *UAS-msi *^*RNAi*^ flies was performed after 100 Gy irradiation. At least 150 flies were used each group with control 100 Gy (−) maintained without RU486, whereas *msi* expression was knocked down in ISCs in 100 Gy (+). (**C**) Relative fold change in the expression of *Diptericin* (*Dpt*) in the dissected gut, 14 days after irradiation. Control 100 Gy (−) maintained without RU486, whereas *msi* was knocked down in ISCs in 100 Gy (+). The error bars indicate S.D. of 4 replicates. (***p < 0.001 by *t*-test). (**D**) Relative fold change in the expression of *Unpaired3* (*Upd3)* in the gut, 24 h after irradiation*.* The error bars indicate S.D. of 4 replicates. (***p < 0.001 by *t*-test). Control 100 Gy (−) maintained without RU486, whereas *msi* was knocked down in 100 Gy (+). (**E**) The number of pH3-positive cells detected per gut of *5961-GS* > *UAS-msi *^*RNAi*^ flies, on day 1 and (**F**) day 14 after irradiation. Control 100 Gy (−) maintained without RU486, whereas *msi* was knocked down in ISCs in 100 Gy (+). The result is represented as mean ± SE of at least 10 guts per group. (^ns^ p > 0.05 by *t*-test) of 3 independent experiments.

Msi belongs to a family of highly conserved RNA-binding translational repressors that are expressed in proliferative progenitor cells^90–92^. To test its role in ISC proliferation upon DNA damage, we used the (RU486)-inducible ISC-specific *5961-GS* driver in 14-day-old adult flies to knockdown *msi* expression^93^. Knocking down *msi* in ISCs (+), followed by irradiation, enhanced the defect in gut permeability by almost two-fold compared to irradiated control flies (without RU486) (−) exposed to 100 Gy (Fig. 5A). We also found that *msi *^*RNAi*^ in ISCs further reduced survival upon irradiation (Fig. 5B). To further characterize the impact of *msi* on radiation sensitivity, we tested whether *msi* expression in ISC’s affected immune activation. Upon irradiation *msi *^*RNAi*^ flies showed a significant upregulation in *Dpt* in the gut (Fig. 5C). Interestingly, the qRT-PCR result in the same dissected guts indicated a reduced expression of *Upd3* upon *msi* knockdown (Fig. 5D).

Because *msi* is known to regulate cell fate and stemness^94^, and irradiation significantly reduces ISC proliferation, we investigated the effect of knocking down *msi* on ISC proliferation in response to radiation. We observed that the ISC proliferation upon knockdown of *msi* in ISCs was relatively lower and comparable to the control flies as measured by immunofluorescence for phospho-Histone 3 (pH3) in dissected guts 1 day and 14 days after irradiation (Fig. 5E,F). ISC-specific *msi* knockdown also reduced ISC proliferation in the un-irradiated control flies, suggesting that *msi* is required for ISC proliferation in general (Supplementary Fig. 4A).

**Figure 6:**
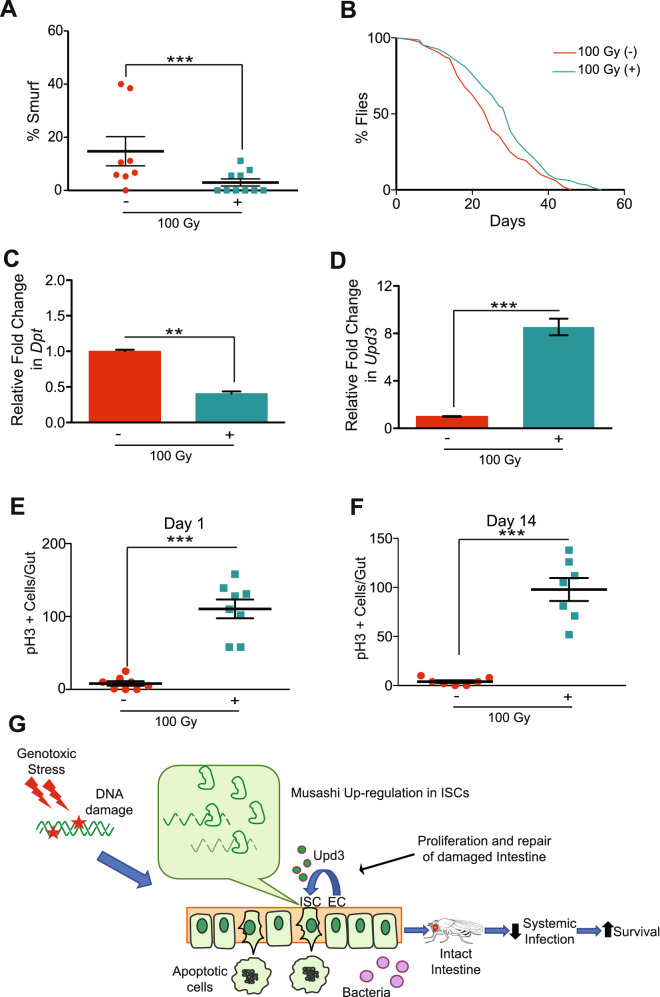
Increasing *msi* expression in ISCs reduces gut permeability and increases survival by restoring stem cell proliferation. (**A**) Smurf assay for assessing gut permeability was performed with *5961-GS* > *UAS-msi* flies on day 14 after irradiation. Control 100 Gy (−) maintained without RU486, whereas *msi* was overexpressed in ISCs in 100 Gy (+). The error bars indicate S.D. of percent Smurf flies per vials. (***p < 0.001 by *t*-test). (**B**) Kaplan Meier survival analysis of *5961-GS* > *UAS-msi* flies was performed after 100 Gy irradiation. At least 150 flies were used each group with control 100 Gy (−) maintained without RU486, whereas *msi* was overexpressed in ISCs in 100 Gy (+). (**C**) Relative fold change in the expression of *Diptericin* (*Dpt*) in the dissected gut, 14 days after irradiation. Control 100 Gy (−) maintained without RU486, whereas *msi* was overexpressed in ISCs in 100 Gy (+). The error bars indicate S.D. of 4 replicates. (**p < 0.01 by *t*-test). (**D**) Relative fold change in the expression of *Unpaired3* (*Upd3)* in the gut, 24 h after irradiation. The error bars indicate S.D. of 4 replicates. (***p < 0.001 by *t*-test). Control 100 Gy (−) maintained without RU486, whereas *msi* was overexpressed in 100 Gy (+). (**E**) The number of pH3-positive cells detected per gut of *5961-GS* > *UAS-msi* flies, on day 1 and (**F**) day 14 after irradiation. Control 100 Gy (−) maintained without RU486, whereas *msi* was overexpressed in ISCs in 100 Gy (+). The result is represented as mean ± SE of at least 10 guts per group. (***p < 0.001 by *t*-test). (**G**) Exposure to ionizing radiation causes DNA damage, that increases apoptosis in the gut, coupled with reduced ISC proliferation. This loss in tissue homeostasis leads to an increase in intestinal permeability, which causes reduced survival perhaps due to systemic infection caused by commensal microbiota. The *msi* over expression in ISCs targets mRNAs like *AC13E* by binding to its 3′UTR, reducing its levels in ISCs which restores stem cell proliferation which not only reduces intestinal permeability but also increases survival.

Consistent with the knockdown analysis, overexpression of *msi* in ISCs (+) in irradiated flies resulted in a significant reduction in gut permeability compared to irradiated control flies (without RU486) (−) (Fig. 6A). Accordingly, *msi* overexpression resulted in a marginal but significant increase in survival and also showed a significant reduction in *Dpt* expression (Fig. 6B,C). However, *msi* overexpression in enterocytes (EC) using *5966-GS* driver did not rescue the gut permeability phenotype in irradiated flies (not shown), which supports an ISC-specific function for *msi*. Interestingly, ISC-specific *msi* overexpression significantly increased *Upd3* expression in the gut 24 h after irradiation (Fig. 6D), as seen by qRT-PCR in dissected intestines. Furthermore, pH3 immunofluorescence staining demonstrated that *msi* overexpression significantly increased ISC proliferation almost 15-fold after irradiation, although overexpression of *msi* in the un-irradiated control flies did not affect ISC proliferation (Fig. 6E,F, and Supplementary Fig. 4B). These results suggest that *msi* overexpression in ISCs re-boosts the regeneration capacity of the intestine after irradiation. Thus, the improved survival of flies upon overexpressing *msi* was, in part, correlated with its ability to increase ISC proliferation. As expected, RU486 on its own did not have any effect on the ISC proliferation (Supplementary Fig. 4C). Next we examined if enhancing ISC proliferation can protect against radiation induced damage. The Cyclin E/CDK2 plays a critical role in the G1 phase and the G1-S phase transition^95^, and when overexpressed, it overcomes cell cycle arrest^96^. We overexpressed *Cyclin E* (*CycE*) in ISCs (with *5961-GS*) to test whether forced ISC proliferation rescued intestinal damage after irradiation. Flies where *CycE* was overexpressed (+), in an ISC-specific manner, had significantly increased ISC proliferation (as measured by pH3 staining in the intestine) when compared to the irradiated control without the RU486 (−) (Supplementary Fig. 5A). However, the overexpression of *CycE* in ISCs had no effect on ISC proliferation in un-irradiated flies (Supplementary Fig. 5B). We then determined whether *CycE* over-expression in ISCs of irradiated flies also reduced intestinal permeability. We performed the Smurf assay in these flies and found a two-fold reduction in the percentage of flies with permeable guts, compared to the irradiated control (Supplementary Fig. 5C). Thus, overexpression of either *msi* or *CycE*, increases ISC proliferation and protects against radiation-induced intestinal permeability.

Msi is an RNA binding protein that modulates the expression of target genes post-transcriptionally by binding to a consensus sequence called *Musashi* Binding Element (MBE) in the 3′UTR of target mRNAs^97^. Hence, to understand the mechanism by which *msi* modulates the ISC proliferation in response to radiation, in silico analysis was performed to identify MBE sites in the 3′UTR of *Drosophila* genes using RBPmap, a web resource developed to identify regulatory RNA motifs and functional sites^98^. Genes with 4 or more binding sites in the 3′UTR were shortlisted (Table S1), and the Smurf assay was performed to test if their knockdown in ISCs would rescue intestinal permeability, amongst the candidates tested (Supplementary Fig. 6A), *Ac13E* knockdown significantly reduced intestinal permeability measured by Smurf assay 14 days after irradiation (Supplementary Fig. 6B).

As the In silico analysis identified MBE sites in the 3′UTR of *Drosophila* genes using RBPmap identified 4 repeats of MBE sites in the *Ac13E* 3′UTR (Supplementary Fig. 6C). We performed RIP-ChIP analysis to determine whether Msi physically interacted with the *Ac13E* 3′UTR. Since our *msi* overexpression fly strain was tagged with HA, we pulled down the mRNAs bound to Msi using anti-HA antibody. The RIP-associated sequences were detected by qRT-PCR using primers encompassing the predicted MBE sites in the 3′UTR of *Ac13E,* and a three-fold enrichment was observed compared to control flies (without RU486) (−) (Supplementary Fig. 6D). We hypothesized that knockdown of the candidate gene, *Ac13E,* would recapitulate *msi* overexpression in terms of radiation-induced gut permeability and reparative proliferation. While *Ac13E* knockdown in ISCs significantly increased ISC proliferation 14 days after irradiation (Supplementary Fig. 5E), it had no effect on ISC proliferation (Supplementary Fig. 5F) in non-irradiated flies.

Finally, our data suggest the model (Fig. 6G) showing that tissue damage caused by genotoxic stress leads to increased apoptosis of the intestinal enterocytes as well as ISCs and, coupled with lack of ISC proliferation, which increased intestinal permeability, and reduced survival perhaps due to systemic infection caused by commensal microbiota. The ectopic expression of *msi* in ISCs restores stem cell proliferative repair by targeting *Ac13E* mRNA (and possibly other targets) that restores barrier function, thus reducing exposure to commensal microbiota and increased survival.

## Discussion

Understanding the mechanisms involved in tissue homeostasis and repair in response to age-related genotoxic stress is critical for developing therapeutics against the side effects of chemotherapeutic agents. However, the lack of an expedient in vivo model has hampered progress in the field. We have developed adult *Drosophila melanogaster* as a model to study how the interaction between different cells help mount a response to genotoxic stress to maintain tissue homeostasis and repair. We leveraged the conservation of the fly intestine to characterize the effect of ionizing radiation on ISC proliferation and intestinal permeability. Our GWAS analysis in these lines identified *msi* as a potential candidate. Further results showed that the levels of *msi* in ISCs correlated with ISC proliferation and ectopic expression of *msi* in ISC not only reduced intestinal permeability but also increased survival in response to irradiation.

Earlier studies have shown that exposure to radiation in adult female flies affects fecundity and increased chromosomal aberrations in the progeny^99^. However, little is known regarding the long-term effect of ionizing radiation on survival of adult flies. Consistent with previous studies, our results demonstrated that flies were quite resistant to tissue damage caused by ionizing radiation^100,101^. Since, exposing flies to staggered doses of radiation, may be more representative of patients undergoing radiation therapy, we exposed flies to either a staggered (4 doses of 25 Gy every other day) or a single dose of 100 Gy. We found that in both exposure regimes, gut permeability was enhanced, and survival was reduced. The effect of radiation on survival was independent of sex as results were consistent between male and female flies.

Since we exposed whole flies to radiation, we expected a strong physiological readout that might explain the shortened survival of irradiated flies. We observed a consistent increase in the phosphorylation of γ-H2Av, the fly orthologue of H2AX^62^. We also observed elevated intestinal permeability and smaller intestines. As increased gut permeability has previously been associated with reduced survival^55,102^ due to increased local and systemic inflammation, we performed the Smurf assay, which demonstrated that irradiated flies have highly permeable intestines. In addition, we observed elevated inflammation in the intestine quite early after irradiation, followed by increased systemic inflammation that temporally correlated with increased intestinal permeability (by day 14 after irradiation). Our results are consistent with previous observations that increased intestinal permeability leads to increased risk of mortality due to bacterial infection^55^.

Importantly, leaky gut syndrome is a hallmark of radiation enteritis in human patients undergoing radiation therapy^103,104^. In humans, the detrimental responses to radiation treatment vary greatly^103,104^ and survival, health, and gut homeostasis may at least in part be regulated by genetic factors^80,83,84^. Thus, we reasoned that fully sequenced natural variants from the DGRP collection would identify novel genes that could restore intestinal homeostasis in irradiated flies. Interestingly, we observed a significant decline in proliferating ISCs, which reduced ISCs numbers in the irradiated flies. The reduced ability of fly ISCs to proliferate in response to radiation-induced damage in the gut is similar to that observed in mammals^32–35^ and even patients receiving radiation or chemotherapy^105^. So, we reasoned that the dual effect of radiation on increased apoptosis in the intestine and reduction in reparative proliferation might be responsible for increased intestinal permeability in irradiated flies. We determined if forcing the restoration of ISC populations might have a protective effect in irradiated flies. In flies, *Cyclin E* alone is capable of activating re-entry into S-phase and promoting ISC proliferation^106^. In addition, overexpression of *Cyclin E* promotes proliferation in cells^96^. Our results confirmed that over-expression of *CycE* in ISCs not only promoted ISC proliferation but also improved intestinal barrier function.

Our studies identified *msi* as one of the modulators of ISC proliferation in response to radiation. Msi, a highly conserved RNA binding protein, is a regulator of post-transcriptional processing of target genes^97^, as well as a known stem cell marker^94^. It was first identified as a regulator of asymmetric division sensory organ precursor cells in *Drosophila*^107^. We found that modulating *msi* in ISCs affected ISC proliferation, which is consistent with the human orthologue, *msi1* that is strongly expressed in the intestinal crypts, especially during embryonic development and regeneration^91^. Interestingly, *msi* overexpression did not significantly impact survival in non-irradiated flies. The stem cell-specific role of *msi* was further confirmed since its ectopic expression in enterocytes had no effect on intestinal permeability. Interestingly, *msi1* knockdown in U-251 (human glioblastoma cell line) resulted in higher instances of double-stranded breaks^108^, suggesting its role in DNA repair. Another study in mice demonstrated that *msi1* and *msi2* could regulate stem cell activation and self-renewal of crypt base columnar cells upon tissue damage, thus indicating a conserved effect of *msi* on ISC function^109^, none the less our findings in conjunction to these reports point to a critical role of Musashi in regenerative medicine.

Musashi regulates target genes by binding to the 3′UTR of its target. It has previously shown to regulate *simA* however we did not observe any protective effect of *simA* knockdown on intestinal permeability in irradiated flies (Supplementary Fig. 6A). However, our results show targeting of *Ac13E* is post transcriptionally regulated by Msi. The Ac13E is an Adenylate cyclase (DAC9) that catalyzes the synthesis of cAMP from ATP, yielding diphosphate as a by-product and its human homologue is ADCY9^110^. It has previously been shown to be involved in elementary associative learning and is responsive to Ca^2+^/Calmodulin^111^. Its role in intestinal permeability is not known, however the increased levels of its human homologue (Adenylyl Cyclase 9) is considered as a prognostic marker in patients with colon cancer^112^. In addition, cyclic AMP produced in the enteroendocrine cells has been shown to be essential for ISC quiescence in *Drosophila* intestine^113^. Our results demonstrate that *Musashi* binds to and regulates *Ac13E* expression. In addition, knocking down *Ac13E* in the ISCs not only increased the ISC proliferation, it also significantly reduced intestinal permeability, thus suggesting a novel regulatory pathway. Taken together, we propose that that Musashi restores intestinal barrier function by enhancing ISC proliferation and tissue repair in response to radiation by targeting *Ac13E* mRNA (Fig. 6G).

## Methods

### Fly culture, stocks and lifespan analysis

Flies were reared on standard laboratory diet (Caltech food recipe: 8.6% Cornmeal, 1.6% Yeast, 5% Sucrose, 0.46% Agar, 1% Acid mix)^114,115^. Eclosed adults were transferred within 3–5 days to the yeast extract (YE) diet (1.5% YE, 8.6% Cornmeal, 5% Sucrose, 0.46% Agar, 1% Acid mix). For *Gene-Switch Gal4* drivers, RU486 was dissolved in 95% ethanol with a final concentration of 100 μM (the media is then referred to as ‘+RU486’) and was administered from the adult stage (5 day old). The control diet contained the same volume of 95% ethanol and is referred to as '−RU486’. Life spans were analyzed as described previously^114,116^. At least 150 flies were used for the life span analysis. The following fly strains were obtained from Bloomington *Drosophila* stock center: *UAS-msi *^*RNAi*^ (BL55152), *UAS-Ac13E *^*RNAi*^ (BL62247), *UAS-CycE* (BL4781). *UAS-msi-HA* (F004549) was obtained from FlyORF. *UAS-CD8-PARP-Venus* was a gift from Dr. Masayuki Miura, *5961-GS* and *5966-GS* from Dr. Heinrich Jasper, *esg-Gal4, UAS-GFP* from Dr. Shigeo Hayashi, *upd3-Gal4* from Dr. Norbert Perrimon.

### Radiation exposure

Adult, female, 5-day old flies were exposed to different doses of X-rays at 320 kV and 10 mA to achieve the required doses as indicated and maintained on a standard fly diet.

### Quantitative real-time-PCR

Total RNA was extracted from at least 12 female guts, 8 female fat bodies (fly abdomen) or 5 female whole flies using Quick-RNA MiniPrep Kit (Zymo Research) per preparation. The cDNA was synthesized using QuantiTect Reverse Transcription Kit (QIAGEN) using 1 µg of total RNA per sample. The qPCR reaction was performed in duplicate on each of at least 3 independent biological replicates using SensiFAST SYBR No-ROX Kit (BIOLINE). Error bars indicate standard deviation. Samples were normalized with *ribosomal protein 49* (*rp 49*).

### Primer sequences

*rp 49*-F: 5′-CCACCAGTCGGATCGATATG-3′

*rp 49*-R: 5′-CACGTTGTGCACCAGGAACT-3′

*Diptericin*-F: 5′-GGCTTATCCGATGCCCGACG-3′

*Diptericin*-R: 5′-TCTGTAGGTGTAGGTGCTTCCC-3′

*Drosomycin*-F: 5′-GAGGAGGGACGCTCCAGT-3′

*Drosomycin*-R: 5′-TTAGCATCCTTCGCACCAG-3′

*hid*-F: 5′-CGATGTGTTCTTTCCGCACG-3′

*hid*-R: 5′-TGCTGCCGGAAGAAGTTGTA-3′

*reaper*-F: 5′-CATACCCGATCAGGCGACTC-3′

*reaper*-R: 5′-ACATGAAGTGTACTGGCGCA-3′

*puckered*-F: 5′-CGGGAACGGGGTAAATCCAA-3′

*puckered*-R: 5′-GAGCAGTTACTACCCGCCAG-3′

*upd3*-F: 5′-ACCTACAGAAGCGTTCCAG-3′

*upd3*-R: 5′-GGTTCTGTAGATTCTGCAGG-3′

### Immunohistochemistry and histology

Immunohistochemistry and histology were performed using protocol previously described^117,118^. For immunohistochemistry, flies were dissected in PEM (100 mM Pipes, 2 mM EGTA and 1 mM MgSO4). Dissected guts were fixed with 4% formaldehyde in PEM for 45 min. Samples were washed for 10 min three times with PEM then incubated with 1% NP40/PEM for 30 min. Samples were washed for 10 min three times with TBS-TB (20 mM Tris–HCl, 130 mM NaCl, 1 mM EDTA, 0.1% Triton X-100 and 0.2% BSA) and blocking was performed with 5% goat serum in TBS-TB for 2 h at room temperature. Samples were incubated with primary antibody overnight at 4 °C, were then washed for 10 min three times with TBS-TB and incubated with secondary antibody for 2 h at room temperature. Nuclei were stained using DAPI. Samples were mounted with Mowiol mounting buffer and analyzed by fluorescence microscope (KEYENCE: BZ-X710). Images were taken at the posterior midgut (region R4). The following antibodies were used in this study: anti-rabbit GFP (Life technologies: 1/500), anti-rabbit phospho-histone H3 (Millipore: 1/1,000), anti-mouse β-galactosidase (Promega: 1/250), anti-mouse γ-H2Av (DSHB: 1/500), anti-rabbit Cleaved PARP (Cell Signaling Technology: 1/100), anti-rabbit Alexa fluor 488 (Cell Signaling Technology: 1/500), anti-mouse Alexa fluor 555 (Cell Signaling Technology: 1/500), anti-rabbit Alexa fluor 555 (Cell Signaling Technology: 1/500) and anti-mouse Alexa fluor 647 (Invitrogen: 1/500).

For histology, flies were washed in 70% ethanol solution for 1 min. Heads without proboscis were fixed in fresh Carnoy’s fixative (ethanol: Chloroform: acetic acid at 6:3:1) overnight at 4 °C. They were then consecutively washed at RT with 30, 50, 70, 90 and 100% EtOH for 10 min each; following which they were transferred to methyl benzoate (MB) for 30 min at RT and to MB: paraffin at 1:1 ratio for 1 h at 65 °C. Heads were then incubated twice for 1 h at 65 °C in melted paraffin and embedded in paraffin blocks. The blocks were sectioned at a thickness of 5 nm, subjected to hematoxylin and eosin staining, and examined by brightfield microscopy.

### Acridine orange staining

Dissected guts were incubated with Ethidium Bromide and acridine orange (Sigma: 5 µg/ml) (10 µg/ml) in PBS for 5 min at room temperature. Samples were rinsed with PBS twice, then mounted with PBS and immediately analyzed by microscope (Olympus: BX51).

### SYTOX orange nucleic acid staining

Dead cells were observed by SYTOX staining as previously described^117^. Dissected guts were incubated with SYTOX Orange Nucleic Acid Stain (Invitrogen: 1 μM) and Hoechst 33342 (Invitrogen: 10 μg/ml) in PEM for 10 min at room temperature. Samples were rinsed with PEM twice, then mounted with PEM and immediately analyzed by microscope (KEYENCE: BZ-X710).

### Smurf gut permeability assay

We performed the assay as previously described with slight modifications^55^. Briefly, 25 flies were placed in an empty vial containing a piece of 2.0 cm × 4.0 cm filter paper. 300 µl of blue dye solution, 2.5% blue dye (FD&C #1) in 5% sucrose, was used to wet the paper as a feeding medium. Smurf and non-smurf flies were counted following incubation with feeding paper for 24 h at 25 °C. Smurf flies were quantified as flies with any visible blue dye outside of the intestines.

### Spontaneous activity

24 h after irradiation, flies (four vials per group with 25 flies in each vial) were placed in population monitors and their physical activity was recorded every 10 min for 24 h (*Drosophila* population monitor by Trikinetics Inc., Waltham, MA, USA). Reading chambers have circular rings of infrared beams at three different levels, which allow recording whenever a fly crosses the rings. Activity monitors were kept in temperature-controlled incubators set at 25 °C on a 12-h light–dark cycle. The daylight period began at 8:00 a.m.

### Screening for variants associated with regulating irradiation-induced phenotypes

We preformed Genome-Wide Association Study as previously described by Nelson et al.^119^**.** Briefly**,** 2 weeks following irradiation, we observed significant variation in the gut permeability Smurf assay between DGRP lines in the proportion of Smurf flies. Candidates with a false detection rate (FDR) of 27% or less were considered for further validation*.* FDRs were calculated empirically from permuted data^89^. The association was determined by aligning phenotypic values at an allelic marker. Genetic markers with > 25% minor allele frequency were used by employing custom scripts written in Python, using ordinary least squares regression from the stats models module^88^. The analysis was done using linear model: phenotype = **β**_1_xGenotype + **β**_2_xIrradiationDose + **β**_3_xGenotype X-Irradiation Dose + intercept. The p-values shown reflect whether the **β** term is 0. The Genotype X-Irradiation Dose term reflects the Irradiation-dependent portion of genetic influence on the phenotype^88^.

## Supplementary information

Supplementary Figure Legends.

Supplementary Figure S1.

Supplementary Figure S2.

Supplementary Figure S3.

Supplementary Figure S4.

Supplementary Figure S5.

Supplementary Figure S6.

Supplementary Table S1.

## Data Availability

The data that supports the findings of this study are available in the supplementary material of this article.
