# Supplementary material for: Musashi expression in intestinal stem cells attenuates radiation-induced decline in intestinal permeability and survival in Drosophila
Source: Sci Rep. 2020 Nov 5;10:19080. doi: 10.1038/s41598-020-75867-z (PMC7644626; doi:10.1038/s41598-020-75867-z)
Supplement: Supplementary file 1 — Supplementary Figure Legends. [file 41598_2020_75867_MOESM1_ESM.docx]

***Musashi* expression in intestinal stem cells attenuates radiation-induced decline in intestinal permeability and survival in *Drosophila***

Amit Sharma*^, †, 3^, Kazutaka Akagi*^, †, 2^, Blaine Pattavina^1^, Kenneth A. Wilson^1^, Christopher Nelson^1^, Mark Watson^1^, Elie Maksoud^1^, Ayano Harata^2^, Mauricio Ortega^1^, Rachel B. Brem^1^, Pankaj Kapahi*^,^ ^1^

^1^Buck Institute for Research on Aging, 8001 Redwood Boulevard, Novato, CA 94945, USA

^2^National Center for Geriatrics and Gerontology, 7-430 Morioka-cho, Obu, Aichi 474-8511, Japan

^3^SENS Research Foundation, 110 Pioneer Way, Suite J, Mountain View, CA 94041, USA

^†^Co-first author

**Contact**

*Correspondence and requests for materials should be addressed to P.K. (pkapahi@buckinstitute.org), A.S. (amit.sharma@sens.org) and K.A. (kazuakg@ncgg.go.jp)

**Supplementary data:**

**Supplementary Figure S1.** **Radiation-induced damage results in a significant reduction in survival and increased intestinal permeability in a dose-dependent manner. (A)** Kaplan Meier survival analysis of female *w^1118^* flies after different doses of irradiation. At least 150 flies were used for each dose. **(B)** Kaplan Meier survival analysis of male *w^1118^* flies, showing that the effect of radiation on survival is independent of sex. At least 150 flies were used for control (0 Gy) and flies irradiated with 100 Gy. **(C)** Representative image of the dissected intestine of adult female fly non-irradiated (0 Gy) control flies and irradiated (100 Gy) female flies 14 days after irradiation. **(D)** The Smurf assay was performed to investigate the effect of radiation on gut permeability in *w^1118^* female flies after irradiation with or without 5 Gy, 10 Gy, 20 Gy and 50 Gy (as indicated) at 14 days after irradiation. Error bars indicate S.D. of 4 replicates. (** p < 0.01, ^ns^ p > 0.05 by *t*-test). **(E)** The Smurf assay was performed to examine the effect of radiation on gut permeability. The adult female *w^1118^* 5 days old adult flies were irradiated with 100 Gy or flies received ‘staggered’ dosage of 25 Gy every other day for 4 days (100 Gy (S)) and compared to non-irradiated control (0 Gy). Smurf assay was performed 14 days after first dose and results were plotted as mean percentage of ‘Smurf’ to non-smurf flies. Error bars indicate S.D. of 4 replicates. (*** p < 0.01, ** p < 0.01 by *t*-test). **(F)** The Smurf assay was performed to investigate the effect of radiation on gut permeability in either male or female flies 14 days after irradiation (100 Gy) and percentage of Smurf flies were compared to non-irradiated control (0 Gy). Smurf assay was performed 14 days after first dose and results were plotted as mean percentage of ‘Smurf’ to non-smurf flies. Error bars indicate S.D. of 4 replicates. (*** p < 0.001 by *t*-test). (G) *Canton-S* flies were maintained in standard fly food and Smurf assay was performed in female flies, 14 days after irradiation (100 Gy) to test the effect on intestinal permeability at day 14. Error bars indicate ± SD (** p < 0.01 by t-test). Each dot represents percent Smurf flies per 25 flies.

**Supplementary Figure S2**. **Quantitation of γ-H2Av, Upd3 and apoptosis in the intestine in response to irradiation. (A)** Quantification of the number of γ-H2Av positive cells from the total number of cells in the image. **(A’)** Quantification of the number of γ-H2Av positive cells from the *esg*-GFP positive cells. Error bars indicate ± SD (** p < 0.01, *** p < 0.001 by t-test). Each dot represents one sample. **(B)** Quantification of the number of nuclear localized upd3 positive cells. Error bars indicate ± SD (*** p < 0.001, ^ns^ p > 0.05 by t-test). Each dot represents one sample. **(C)** Quantification of the number of ISCs undergoing apoptosis. Error bars indicate ± SD (*** p < 0.001 by t-test). Each dot represents one sample. **(D)** Midguts were stained with SYTOX orange to detect dead cells. Guts from *esg-Gal4, UAS-GFP, Dl-LacZ, UAS-CD8-PARP-Venus* flies were dissected 2 day after irradiation with (bottom panels) or without (top panels) 100 Gy. Scale bar indicates 20 μm. **(D’)** Quantification of the number of SYTOX positive cells out of the number of *esg*-GFP negative cells (EC, EE) in the image. Error bars indicate ± SD (* p < 0.05 by t-test). Each dot represents one sample.

**Supplementary Figure S3. Secondary screen for intestinal permeability for candidate genes from DGRP lines by Smurf assay.** The expression of candidate genes was knocked down in ISCs using *5961-GS driver. 5961-GS> UAS-candidate gene RNAi* flies were maintained in standard fly food with RU486 (+) to knockdown its expression or without RU486 (-). Two days after adding RU486, flies were irradiated with 100 Gy to test the effect of its knockdown on intestinal permeability. Results were plotted as mean change in the percentage of flies with permeable guts and the error bars indicate S.D. of 4 replicates.

**Supplementary Figure S4. Modulating *msi* expression in ISCs did not affect stem cell proliferation in non-irradiated flies.** The expression of genes modulated in ISCs using *5961-GS driver*, the rate of ISC proliferation was quantified without irradiation (0 Gy)*.* Flies with **(A)** *msi ^RNAi^* and **(B)** *Msi* overexpression (*UAS-msi*) were maintained in standard fly food with (+) to modulate its expression or without RU486 (-). Immunostaining with anti-pH3 antibody was performed to test the effect of gene modulation on ISC proliferation at day 14. Error bars indicate ± SD (** p < 0.01, ^ns^ p > 0.05 by t-test). Each dot represents one sample. **(C)** The number of pH3-positive cells detected per gut of *5961-GS> UAS-GFP* flies, on day 14 after irradiation. Flies were maintained in standard fly food with (+) or without RU486 (-). The error bars indicate ±SD of at least 16 guts per group. (*** p < 0.001, ** p < 0.01, ^ns^ p > 0.05 by *t*-test).

**Supplementary Figure S5. Increasing *Cyclin E* expression in ISCs reduces gut permeability by restoring stem cell proliferation after irradiation. (A)** Number of pH3-positive cells detected per gut of *5961-GS> UAS-CycE* flies irradiated with 100 Gy. Guts were dissected and pH3 staining was performed after 14 days of irradiation. Controls 100 Gy (-) maintained without RU486, whereas *CycE* was overexpressed in ISCs in 100 Gy (+). **(B)** Number of pH3-positive cells detected per gut of unirradiated *5961-GS> UAS-CycE* flies (0 Gy). Guts were dissected and pH3 staining was performed. Controls 0 Gy (-) maintained without RU486, whereas *CycE* was overexpressed in ISCs in 0 Gy (+). The result is represented as mean ±SD of 14 guts per group. (*** p < 0.001, ** p < 0.01 by *t*-test). **(C)** Smurf assay for assessing gut permeability was performed with *5961-GS> UAS-CycE* flies on day 14 after irradiation. Control 100 Gy (-) maintained without RU486, whereas *CycE* was overexpressed in ISCs in 100 Gy (+). Results plotted as mean proportion of Smurf to non-Smurf flies of in each group with 25 flies in each vial. Error bars indicate SEM. (*** p < 0.001 by *t*-test).

**Supplementary Figure S6. The expression of candidate target genes with binding site for the Msi in their 3’UTR were knocked down in ISCs using *5961-GS driver.*** *5961-GS> UAS-candidate gene* flies were maintained in standard fly food with (+) to knockdown its expression or without RU486 (-). Two days after adding RU486, flies were irradiated with 100 Gy to test the effect of its knockdown on intestinal permeability was performed. **(A)** The results of the Smurf assay were plotted as mean change in the percentage of flies with permeable guts and the error bars indicate S.D. of 4 replicates. (** p < 0.01, * p < 0.05 by *t*-test). **(B)** Smurf assay of *5961-GS> UAS-Ac13E RNAi* flies was performed at 14 days after 100 Gy irradiation*.* Control 100 Gy (-) flies were maintained without RU486, whereas *Ac13E* was reduced in ISCs in 100 Gy (+). The error bars indicate S.D. of percent of Smurf flies per vials. (*** p < 0.001 by *t*-test). **(C)** *In silico* analysis to identify MBE sites in the 3’UTR of *Drosophila* genes using RBPmap identified 4 repeats of MBE sites in the 3’UTR. **(D)** Musashi targets mRNA for *Ac13E* mRNA, RIP-ChIP analysis is performed using *5961-GS> UAS-msi-HA* flies were maintained with (+) or without RU486 (-) for 7 days followed by dissection of the guts. Cell lysates were incubated with Dynabeads-anti-HA to pull down mRNAs bound to msi-HA complex. RNA was precipitated with phenol/chloroform, and isopropanol. Quantitative real-time PCR was performed to detect the binding of 3 different regions *Ac13E* 3’UTR (predicted target of msi) as described before and data was analyzed using 2^ΔΔCt^method. **(E)** The number of pH3-positive cells per gut were detected in *5961-GS> UAS-Ac13E RNAi* flies on 14 days after 100 Gy irradiation. The result is represented as mean ±SE of at least 10 guts per group. (*** p < 0.001 by *t*-test) of 3 independent experiments. **(F)** The number of pH3-positive cells per gut were detected in *5961-GS> UAS-Ac13E RNAi* flies on 14 days without irradiation (0 Gy). Error bars indicate ± SD (^ns^ p > 0.05 by t-test). Each dot represents one sample.

**Supplementary Table 1. A ranked list of transcripts predicted to be bound by *Musashi*.** PSAM affinity model across all 3' UTRs annotated in Flybase, and then cut off the affinity score at 0.75 of the optimal predicted affinity were included as positive hit.

**Supplementary Methods:**

**Prediction of genes with Msi binding site**

We used PASM affinity model described by Foat *et al.* (Foat, Tepper, & Bussemaker, 2008) to predict Musashi binding element sequence. The conserved three-nucleotide core motif that determines musashi-binding specificity (Zearfoss et al., 2014) was predicted to bind in the 3’UTRs sequences annotated in the mRNAs obtained from the flybase.org. The cut off the affinity score at 0.75 was set for the optimal predicted affinity.

**RIP-ChIP analysis**

RIP-ChIP analysis was performed to determine the mRNA bound to the *musashi* using protocol describe by Vo *et al* (Vo et al., 2012). Briefly, *5961-GS> UAS-msi-HA* flies were maintained with or without RU486 for 7 days followed by dissection of the guts. Cell lysates were prepared in polysome lysis buffer (100 mmol/L KC, 25 mmol/L EDTA, 5 mmol/L MgCl_2_, 10 mmol/L HEPES, pH 7.0, 0.5% Nonidet P-40, 10% glycerol, 2 mmol/L dithiothreitol, 0.4 mmol/L vanadyl ribonucleoside complex, one tablet of complete protease inhibitor (Roche Applied Sciences, USA) and centrifuged, and the supernatant was removed and stored at −80°C. Protein A Dynabeads beads (Thermo Fisher Scientific, USA) were incubated overnight at 4°C with either anti-HA1 polyclonal antibody (Roche, USA). The cell lysate was added, and the mixture of beads and lysate was incubated at room temperature while tumbling end over end for 4 hours. After incubation, the beads were collected by using magnetic columns and washed two times with Washing Buffer (with 1x RNAse buffer and 1x protease inhibitor) and then twice by ice-cold PBS buffer. Dynabeads-Ab-Ag complex was re-suspended, and the suspension was transferred   to a clean tube.  Subsequently, the material was digested with proteinase K, extracted with phenol/chloroform, and precipitated with isopropanol. Quantitative real-time PCR was performed as described before and data was analyzed using 2 ^ΔΔCt^ method.
